# Scalable fabrication of self-assembled GeSn vertical nanowires for nanophotonic applications

**DOI:** 10.1515/nanoph-2022-0489

**Published:** 2023-01-12

**Authors:** Guangyang Lin, Yuying An, Haokun Ding, Haochen Zhao, Jianyuan Wang, Songyan Chen, Cheng Li, Ryan Hickey, James Kolodzey, Yuping Zeng

**Affiliations:** Department of Physics, Xiamen University, Xiamen, Fujian 361005, People’s Republic of China; Department of Electrical and Computer Engineering, University of Delaware, Newark, DE 19716, USA

**Keywords:** dry etching, germanium tin, near infrared, photodetector, self-assembled nanowire

## Abstract

In this work, scalable fabrication of self-assembled GeSn vertical nanowires (NWs) based on rapid thermal annealing (RTA) and inductively coupled-plasma (ICP) dry etching was proposed. After thermal treatment of molecular-beam-epitaxy-grown GeSn, self-assembled Sn nanodots (NDs) were formed on surface and the spontaneous emission from GeSn direct band was enhanced by ∼5-fold. Employing the self-assembled Sn NDs as template, vertical GeSn NWs with a diameter of 25 ± 6 nm and a density of 2.8 × 10^9^ cm^−2^ were obtained by Cl-based ICP dry etching technique. A prototype GeSn NW photodetector (PD) with rapid switching ability was demonstrated and the optoelectronic performance of Ge NW PD was systematically studied. The GeSn NW PD exhibited an ultralow dark current density of ∼33 nA/cm^2^ with a responsivity of 0.245 A/W and a high specific detectivity of 2.40 × 10^12^ cm Hz^1/2^ W^−1^ at 1550 nm under −1 V at 77 K. The results prove that this method is prospective for low-cost and scalable fabrication of GeSn NWs, which are promising for near infrared or short wavelength infrared nanophotonic devices.

## 1 Introduction

Over the past two decades, the dramatically increasing demand for communication capacity and speed has boosted the development of silicon (Si)-based optoelectronics. Due to high carrier mobilities, high absorption coefficient at telecommunication wavelength, quasi-direct bandgap structure and compatibility with Si processing technology, germanium (Ge) is regarded as one of the promising materials for Si-based optoelectronics [1]. The growth of high-quality Ge films [2–4] and development of high-performance Ge-based optoelectronic devices [5–7] have been extensively demonstrated. Recently, the attention has been paid to germanium tin (GeSn) for its eminent electronic and optoelectronic properties. It has been predicted [8] that GeSn possesses higher hole mobility compared to Ge. High mobility GeSn p-channel metal-oxide-semiconductor field-effect-transistors (FETs) have been extensively demonstrated [9, 10]. As Sn content increases, the bandgap of GeSn can be tuned from near infrared [11] (NIR) to mid infrared [12, 13] (MIR) ranges with enhanced absorption coefficient, rendering GeSn acclaimed for NIR and MIR photodetectors (PDs) [14, 15]. More importantly, GeSn would transform to a direct bandgap material at a Sn content higher than ∼8% [16, 17], which makes GeSn a promising material for monolithic light source on Si. Optically pumped [18–21] and electrically pumped [22, 23] GeSn lasers have been successively demonstrated since 2016.

Low-dimensional structures provide an extra freedom to control the electronic and optoelectronic properties of semiconductors through regulating their size due to the quantum confinement effect. Additionally, the small volume and large surface to volume ratio of the low-dimensional structures, especially nanowires (NWs), facilitate strain engineering [24] of the material and enhance the interaction between light and the material [25]. Numerous studies have been devoted to fabrication of GeSn NWs since 2015. Generally, the fabrication of GeSn NWs can be categorized into two strategies: bottom-up growth and top-down processing. Using Sn as catalyst, GeSn homogeneous NWs have been synthesized by microwave-assisted solution–liquid–solid growth [26]. However, the control of NW orientation, size and distribution face challenge. Choosing Au [27] or AuAg [28] as catalyst, controllable GeSn homogeneous [29] and Ge/GeSn core/shell [24, 30] NWs were prepared by chemical vapor deposition. Yet, the use of noble metals may degrade the optical performance of GeSn through inducing deep energy levels in the bandgap of GeSn, similar to the cases in Ge [31]. Relying on high-resolution electron beam lithography (EBL) and inductively coupled plasma (ICP) dry etching techniques, the top-down fabrication of vertical [32, 33] and horizontal [34] GeSn NWs were reported for fabrication of high-performance gate-all-around (GAA) FETs or Fin-FETs. Nevertheless, the use of expensive EBL hinders the application of this approach in large-scale fabrication. A cost-effective and simple approach to fabricate GeSn NWs with good optical performance is desired.

In this work, we propose scalable fabrication of self-assembled GeSn vertical NWs by MBE growth, rapid thermal annealing (RTA) and subsequent ICP dry etching. The RTA process improves the optical property of the MBE-grown GeSn and enables formation of self-assembled Sn nanodots (NDs) on sample surface simultaneously. Taking the self-assembled Sn NDs as hard mask, vertical GeSn NWs are obtained by Cl-based ICP dry etching process. With the GeSn NWs, a prototype GeSn NW PD is fabricated and the optoelectronic properties are systematically studied. The prototype GeSn NW PD exhibits a responsivity of 0.245 A/W and a specific detectivity of 2.40 × 10^12^ cm Hz^1/2^ W^−1^ at 1550 nm under −1 V at 77 K with rapid switching ability. The results prove that this method is promising for low-cost and scalable fabrication of high-quality GeSn NWs, which have a great potential for NIR or short wavelength infrared (SWIR) nanophotonic devices.

## 2 Experiments and methods

### 2.1 Experiments

#### 2.1.1 Epitaxy of GeSn sample

The GeSn sample was grown on a 3-inch undoped (001)-oriented Ge substrate with a resistivity of 40 Ω·cm by molecular beam epitaxy (MBE) [35]. After wafer cleaning, the substrate was degassed in pretreatment chamber then was transferred to the growth chamber with a base pressure of 3 × 10^−10^ Torr. Prior to GeSn deposition, the substrate was deoxidized at 850 °C for 10 min. The substrate temperature was then lowered to 150 °C for GeSn deposition. The Ge and Sn sources were fed through thermal evaporation of triple zone-refined Ge and Sn (6N, United Mineral and Chemical Corporation) loaded in Knudsen thermal effusion cells, respectively. During growth, the Ge cell and Sn cell temperatures were maintained at 1220 °C and 980 °C, respectively. After a growth period of 3 h, GeSn film with nominal Sn content of 6.6% and nominal thickness of 200 nm was formed.

#### 2.1.2 Fabrication of self-assembled GeSn vertical NWs

The MBE-grown GeSn sample was subjected to RTA by Solaris 150 Rapid Thermal Processing System. The sample was first heated from room temperature to 550 °C with a ramp rate of 120 °C/s in pure N_2_ ambient (5N) then was maintained at 550 °C for 1 min. Finally, the sample was cooled down to <100 °C and taken out from the RTA chamber in 8 min. Based on our previous studies, Sn NDs would form on sample surface after appropriate thermal treatment due to larger surface free energy of Sn than that of Ge. Using the segregated Sn NDs as hard mask, we further explored feasible fabrication of self-assembled GeSn nanostructures by dry etching method. Consequently, the annealed sample was processed with ICP dry etching for 4 min using Cl_2_, Ar and O_2_ as working gas at 10 mTorr, which was explored previously [36]. The ICP power and RF power were set at 200 W and 100 W, respectively. With this approach, GeSn vertical NWs were obtained.

#### 2.1.3 Fabrication of GeSn vertical NW PD

To evaluate the optoelectronic properties of the prepared GeSn vertical NWs, a prototype GeSn NW PD was fabricated. The NW sample was first rinsed in diluted HCl solution to remove residual Sn NDs, then was spun-on with ∼150 nm polymethyl methacrylate (PMMA) to isolate the substrate. Next, descum was conducted by asher to exposure the NW tip followed by deposition of 8 nm-thick TiO_2_ by Savannah S100 atomic layer deposition (ALD) system at 150 °C using H_2_O and tetrakis(dimethylamino)titanium(IV) as precursors. The deposited TiO_2_ naturally acted as a n-type transparent conductive layer [37]. Finally, Al/Ti/Au stack and Ti/Au stack were deposited on TiO_2_ and backside of Ge substrate, respectively, forming ohmic contacts for electrodes. The area of the diode is 2 × 3 mm^2^. Due to unintentional p-doping of GeSn, p-GeSn NWs/n-TiO_2_ diode was fabricated on bulk Ge.

### 2.2 Methods

#### 2.2.1 Characterization of GeSn film and NWs

X-ray diffractor (XRD), atomic force microscope (AFM), scanning electron microscope (SEM), transmission electron microscope (TEM) and X-ray energy dispersive spectroscope (EDS) were employed to characterize the structural properties of GeSn samples. The (004) reflection *ω* − 2*θ* rocking-curve scan and ( $\bar{2}\bar{2}4$ ) reciprocal space mapping (RSM) taken by Panalytical X′ Pert XRD were used to evaluate the crystal quality, Sn content and strain level of GeSn film. The surface morphology of GeSn film was scanned by Anasys NanoIR2 AFM using taping mode. The morphology and distribution of GeSn NWs was examined by Zeiss SIGMA-HD SEM. TEM images and X-ray energy dispersive spectra of GeSn NWs were obtained by FEI Talos F200X to characterize elemental distribution and crystal quality of GeSn NWs.

#### 2.2.2 Measurement of photoluminescence (PL) and response spectra

PL spectra were collected to characterize the optical property of as-grown and annealed GeSn sample. The PL signal of GeSn film was excited by a 1064 ± 2 nm continuous laser with a spot size of ∼120 μm and a power of 530 mW. Monochromatic light was obtained by Princeton Instruments SP2358 monochrometer with a 300 g/mm grating then was collected by an InGaAs PD. Lock-in technique was employed to improve the signal to noise ratio at a reference frequency of 320 Hz. Detailed configuration of the setup is exhibited in Supplementary Material. Temperature-dependent photoresponse of the prototype NW PD was measured to evaluate the optical property of fabricated NWs. The current–voltage (IV) curves the NW PD were taken by Keithley 2611B source meter at 77–296 K. The sample temperature was controlled by Janis CCS-100/204 cryostat. A tungsten halogen lamp was used as light source for photocurrent measurements. The light was first guided into the monochrometer, then was coupled into a 600 μm fiber and finally shined normally on the device. Detail of the setup has been described previously [38]. The light intensity was calibrated by a commercial InGaAs PD.

## 3 Results and discussion

### 3.1 Self-assembled vertical GeSn NWs

**Figure 1:**
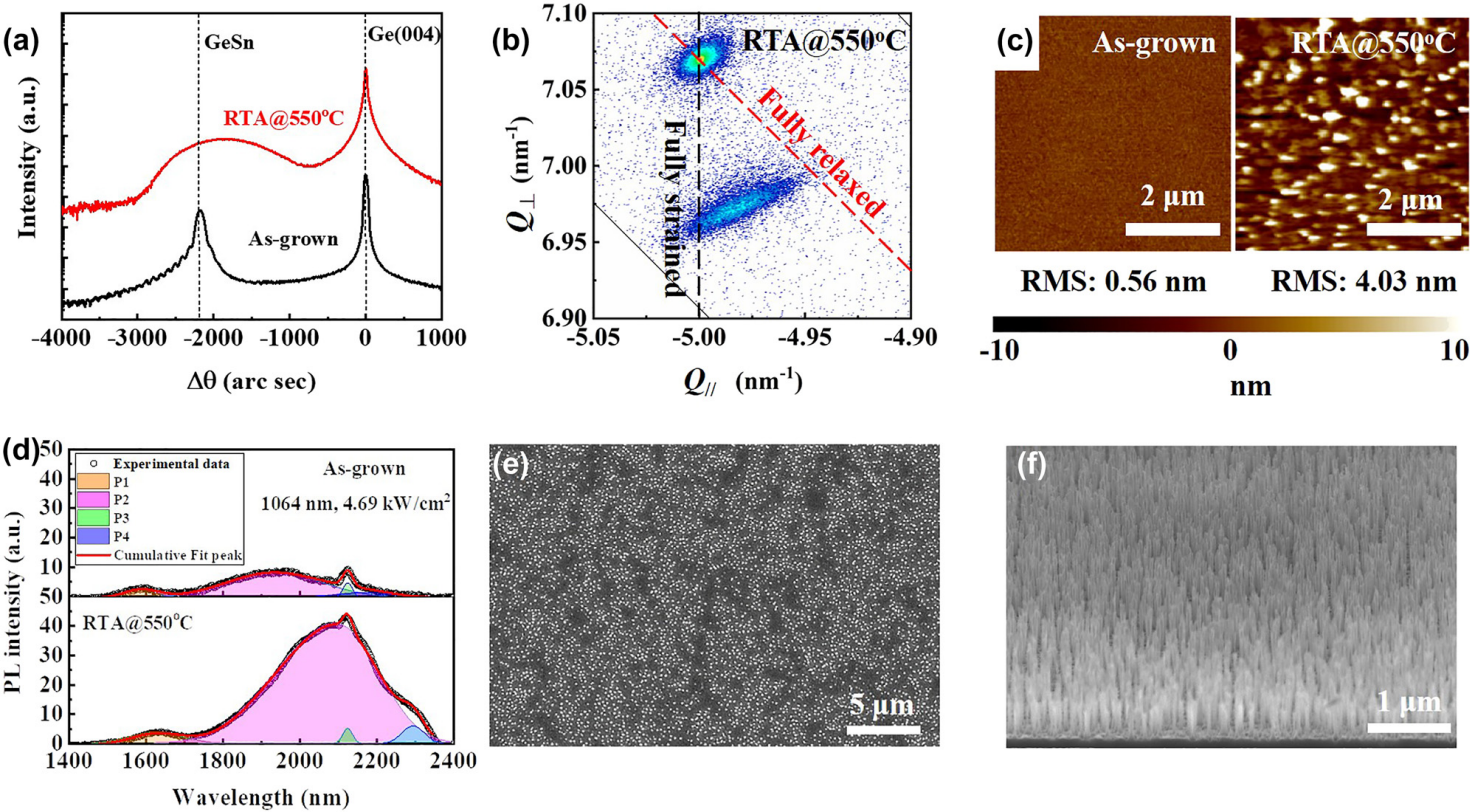
Structural characterization of as-grown GeSn film, annealed GeSn film and GeSn NWs. (a) XRD (004) *ω* − 2*θ* rocking-curve scans of GeSn grown on Ge substrate by MBE before (black curve) and after (red curve) RTA at 550 °C. (b) ( $\bar{2}\bar{2}4$ ) XRD-RSM of GeSn after RTA at 550 °C. (c) AFM images of the GeSn sample before (left panel) and after (right panel) RTA at 550 °C. (d) Room temperature PL spectra of the GeSn sample before (upper panel) and after (lower panel) RTA at 550 °C. (e) Top-view and (f) tilted-view SEM images of the GeSn sample after RTA at 550 °C and subsequent Cl-based ICP dry etching.

The black curve of Figure 1(a) shows XRD (004) *ω* − 2*θ* rocking-curve scan of the as-grown sample. The observation of Pendellösung fringes indicates a sharp and smooth interface between Ge and GeSn and implies that the GeSn film is coherently strained to Ge. Based on the peak separation between Ge and GeSn and the periodicity of the fringes, the Sn content and thickness of the GeSn film were determined to be 6.62% and 200 nm, respectively (see SupplementaryMaterial for detail). The red curve in Figure 1(a) displays XRD (004) *ω* − 2*θ* rocking-curve scan of the sample after RTA. The GeSn peak broadens and shifts to higher angle side, suggesting strain relaxation of the GeSn film. Figure 1(b) further shows the ( $\bar{2}\bar{2}4$ ) XRD RSM of the annealed sample, from which the Sn content and strain relaxation of the GeSn film are calculated to be 6.57% and 31.99%, respectively. Figure 1(c) compares the AFM images of the GeSn sample before (left panel) and after (right panel) the thermal treatment. The as-grown sample exhibits a flat surface with a surface roughness root-mean-square (RMS) of 0.56 nm. After annealing, the surface roughness RMS raised to 4.03 nm. Some NDs were formed on surface, which can be ascribed to Sn segregation and migration [39] on surface during thermal treatment.

Figure 1(d) shows the PL spectra of the GeSn sample before (upper panel) and after (lower panel) RTA measured at room temperature, respectively. The broad PL spectra are originated from the following four contributions [40]: direct bandgap radiation of Ge (P1), direct bandgap radiation of GeSn (P2), 2nd order diffraction of pumping laser by grating fixing at ∼2125 nm (P3) and indirect bandgap emission of GeSn (P4). Detailed analysis of PL spectra can be found in the Supplementary Material. After annealing at 550 °C, the emission wavelength of GeSn direct bandgap shifts from 1934 nm to 2088 nm. Moreover, the PL intensity from GeSn direct bandgap radiation is improved by ∼5-fold. The red shift of emission wavelength and enhanced emission intensity of GeSn direct bandgap can be ascribed to strain relaxation of the GeSn film, which leads to shrinkage of GeSn bandgap and increased directness of GeSn bandgap. The result indicates that although Sn NDs are formed on GeSn surface after annealing, the optical property of GeSn is improved.

Figure 1(e) and (f) exhibit the top-view and tilted-view SEM images of the annealed sample after ICP dry etching, respectively. As can be seen, abundant vertical NWs are obtained. The density of the vertical NWs is evaluated to be 2.8 × 10^9^ cm^−2^. The diameter of the vertical NW is within 25 ± 6 nm. By adjusting the RTA temperature, the NW diameter and density can be adjusted (see Supplementary Material for detail) and will be further studied in future work.

**Figure 2:**
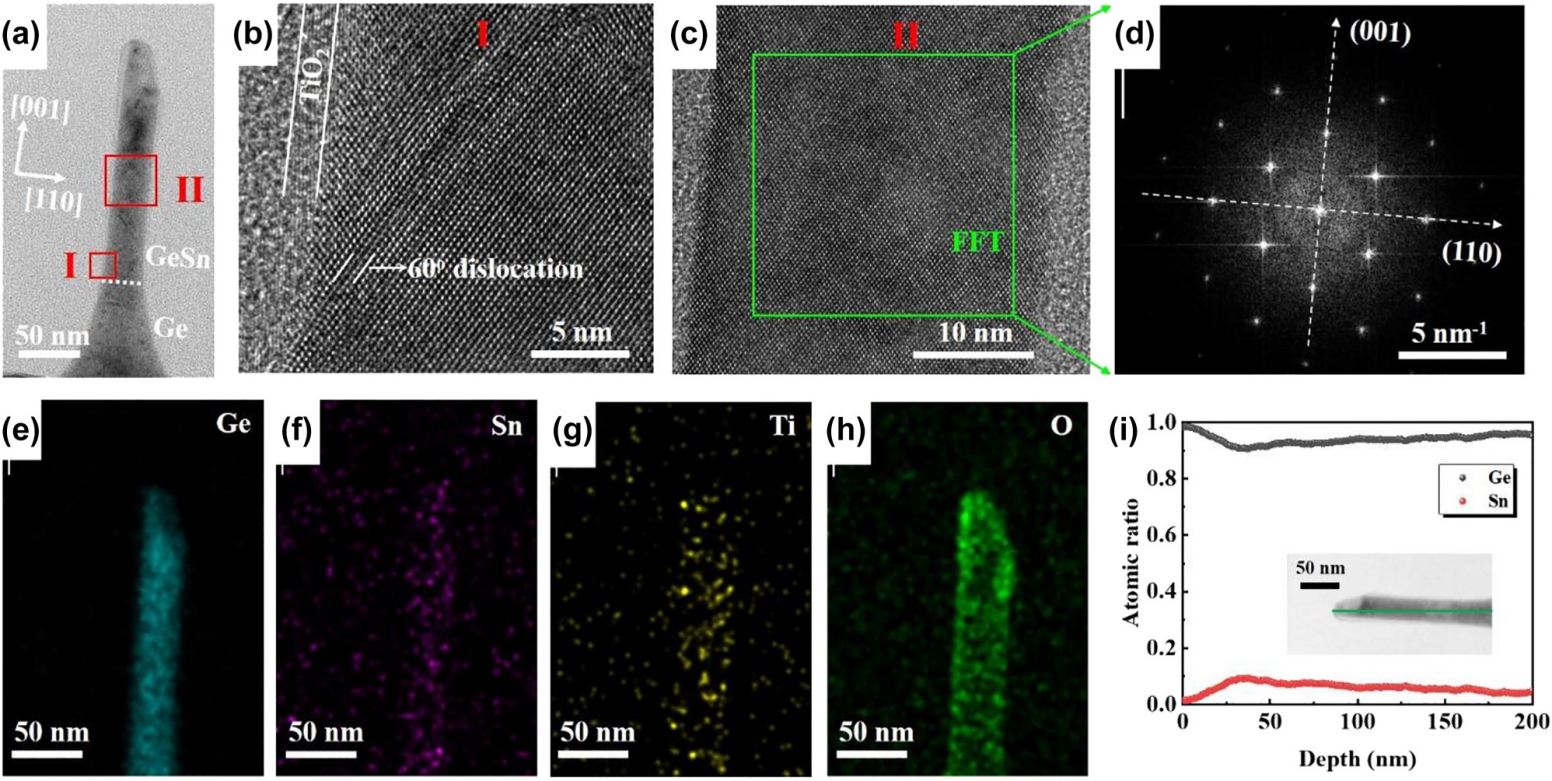
TEM and EDX characterization of a typical GeSn NW. (a) Cross-section TEM image of a GeSn vertical NW coated with 4 nm TiO_2_; HRTEM images of (b) region I and (c) region II in Figure 2(a). (d) FFT of the GeSn NW region in the green square of Figure 2(c). EDX area scans of (e) Ge, (f) Sn, (g) Ti, and (h) O elements of the vertical GeSn NW. (i) Sn and Ge depth profiles of the GeSn vertical NW taken from EDX line scan.

To characterize the crystal quality of the fabricated GeSn NWs, cross-section TEM image of a typical NW is taken, as shown in Figure 2(a). The sample was prepared by focus ion beam (FIB) thinning. To prevent extrinsic damage of the NW induced by the FIB thinning process, a 4 nm-thick TiO_2_ protective layer was coated on the NW by ALD at 150 °C. The total height of the NW is around 292 nm, suggesting that the NW is made up of the initial GeSn region (200 nm) at top and the initial Ge region (92 nm) at bottom. The diameter of initial GeSn region along the NW is uniform with a dimension of ∼30 nm, except that a bevel was formed at the NW tip. For the initial Ge region, the diameter gradually increases from ∼30 nm at initial GeSn/Ge interface to ∼90 nm at NW bottom. Figure 2(b) further displays the high-resolution TEM (HRTEM) image of the NW at GeSn side adjacent to the GeSn/Ge interface (region I in Figure 2(a)). A gliding dislocation along <111> facet is clearly observed, which is formed during RTA process and makes a significant contribution to the strain relaxation of GeSn film. Figure 2(c) presents the HRTEM image taken from the middle area (region II in Figure 2(a)) of NW. Dislocations and defects are absent in this region suggesting good crystal quality of the material. Figure 2(d) shows the fast Fourier transformation (FFT) image of the green square region in Figure 2(c). The diffraction spots from (001) to (110) facets have been identified. The ordered arrangement of diffraction spots suggests good crystal quality of the vertical NW.

Figure 2(e)–(h) display the areal distribution of Ge, Sn, Ti and O elements for the vertical NW taken from EDX, respectively. The decoration of Ti and O elements on the NW sidewall indicates that the NW is well protected by the TiO_2_ during the FIB thinning process. The Ge and Sn elements can also be clearly recognized in the NW verifying acquisition of GeSn NW. Figure 2(i) further shows the Sn and Ge depth profiles along the GeSn NW taken from EDX line scan. At NW top surface, the Sn content is ∼1.5%, which is close to the solid solubility of Sn in Ge and suggests Sn segregation on NW tip. As depth increases, the Sn content gradually enriches to 9.1% at a depth of 31 nm then decreases to 4.3% at a depth of 200 nm. The results indicate that Sn diffuses out from the film and segregates on surface during the RTA process leading to formation of Sn-component-graded (SCG) heterojunction NWs.

**Figure 3:**
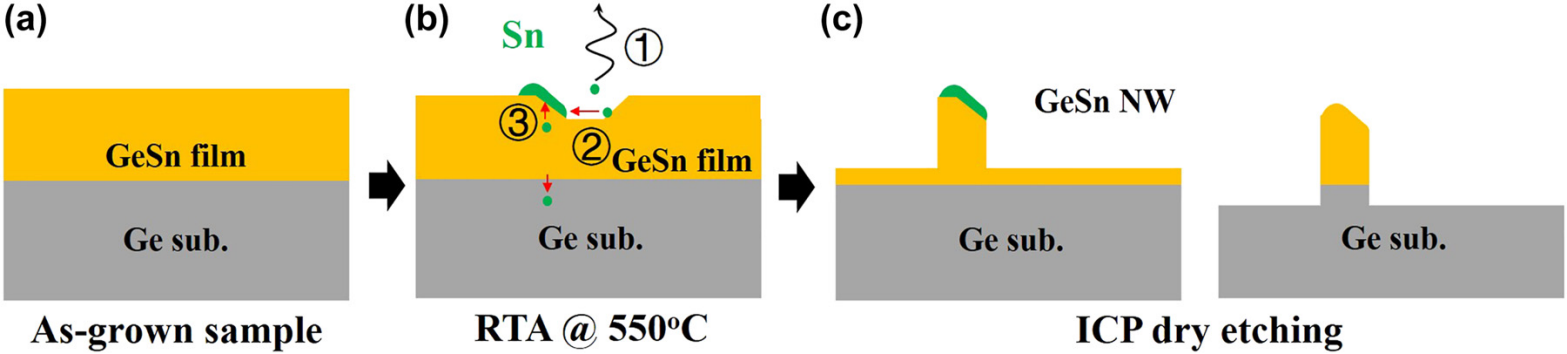
Structure diagram of the sample after each process for fabrication of self-assembled GeSn vertical NWs: (a) as grown sample; (b) annealed sample; (c) NW sample.

The formation of GeSn vertical NW can be depicted in Figure 3 and explained as follows. The surface of initial GeSn sample is flat (Figure 3(a)). During the RTA process, the kinetic energy of Sn atoms increases significantly, which drives Sn desorption (①), migration (②) on surface and outdiffusion (③) from the GeSn/Ge interface toward surface. The desorption and migration of Sn atoms gives rise to formation of nano-slopes on surface [41], where Sn segregated into ND on the slope due to distinct surface-free energies between Sn and Ge, as exhibited in Figure 3(b). As the sample is submitted to ICP dry etching, Sn NDs can act as hard mask. The region without Sn NDs on surface is etched away. As a result, self-assembled vertical GeSn NWs with a bevel at each NW tip are formed due to high etching selectivity of GeSn to Sn, as depicted in left panel of Figure 3(c). Depending on the etching duration, Sn may be also etched away, as shown in right panel of Figure 3(c).

### 3.2 Self-assembled GeSn vertical NW PD

**Figure 4:**
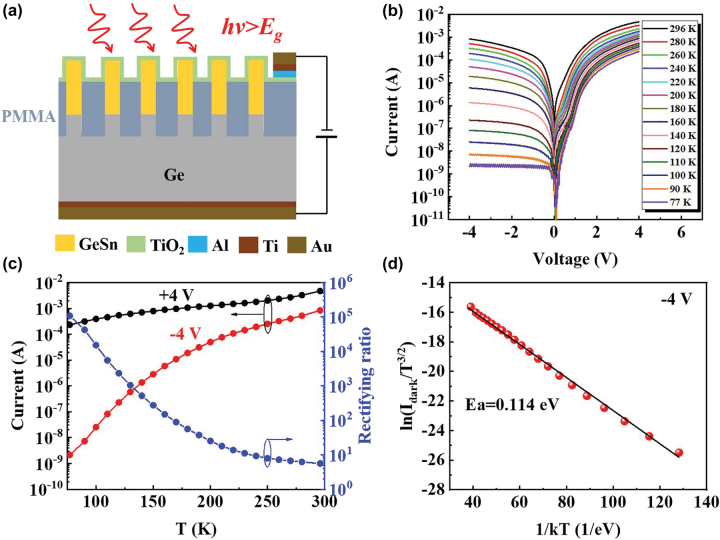
Electrical properties of fabricated GeSn NW PD. (a) Cross-section diagram of the prototype GeSn NW PD. (b) Temperature-dependent *I*–*V* curves of the NW PD under dark at 77–296 K. (c) Dark current under +4 V (black curve), −4 V (red curve) and current rectifying ratio under ±4 V (blue curve) of the NW PD as a function of temperature. (d) Dependence of ln(*I*_dark_/*T*^3/2^) on 1/*kT* and corresponding linear fitting curve.

Figure 4(a) shows the cross-section diagram of the prototype GeSn NW PD. The black and red curves in Figure 4(b) display the *I*–*V* curves of the diode under dark at 77 K–296 K. Figure 4(c) summarizes the dependence of dark current under +4 V (black curve), −4 V (red curve) and the current rectifying ratio under ±4 V (blue curve) for the NW PD on temperature. At 296 K the NW PD exhibits a low current rectifying ratio of 5.6 with a forward current of 4.7 mA under +4 V and a reverse current of 0.83 mA under −4 V. As temperature decreases, the forward current under +4 V gradually decreases from 4.7 mA at 296 K to 0.23 mA at 77 K, while the reverse dark current under −4 V dramatically decreases from 0.83 mA at 296 K to 2.1 nA at 77 K. Consequently, the NW PD presents a high current rectifying ratio of >10^5^@±4 V at 77 K with a dark current of 2.1 nA@−4 V corresponding to a current density of ∼33 nA/cm^2^. The reverse dark current density is ultra-low compared to typical Ge- or GeSn-based devices [42] (typically >mA/cm^2^). To understand the origin of the reverse dark current, the activation energy (*E*_*a*_) of the GeSn NW PD can be extracted for analysis by [43]:

(1) $$I_{\text{dark}}=BT^{3/2}\exp\left(\frac{-E_{a}}{kT}\right)\left[\exp\left(\frac{qV_{b}}{2kT}-1\right)\right],$$

where *V*_*b*_ is reverse bias and *V*_*b*_ = −4 V is used for analysis herein, *I*_dark_ is the dark current at *V*_*b*_, *B* is a constant, *q* and *k* are the elementary charge and Boltzmann constant, respectively. Figure 4(d) shows the plot of ln(*I*_dark_/*T*^3/2^) versus 1/*kT*. Through linear fitting the curve, *E*_*a*_ = 0.114 eV can be obtained from the slope. The activation energy is much smaller than half of the bandgap of GeSn. The result indicates that the reverse dark current is dominated by surface leakage current [43]. High-density surface shallow traps, which can be caused by ICP dry etching process, exist on the NW sidewall. Hence, the GeSn NW PD presents a high reverse dark current at room temperature while an ultrasmall reverse dark current at 77 K due to suppression of Shockley–Read–Hall (SRH) recombination via surface traps at low temperature. Through surface defect removing (such as digital etching [44]) and surface passivation [45] techniques, the reverse dark current can be further suppressed.

**Figure 5:**
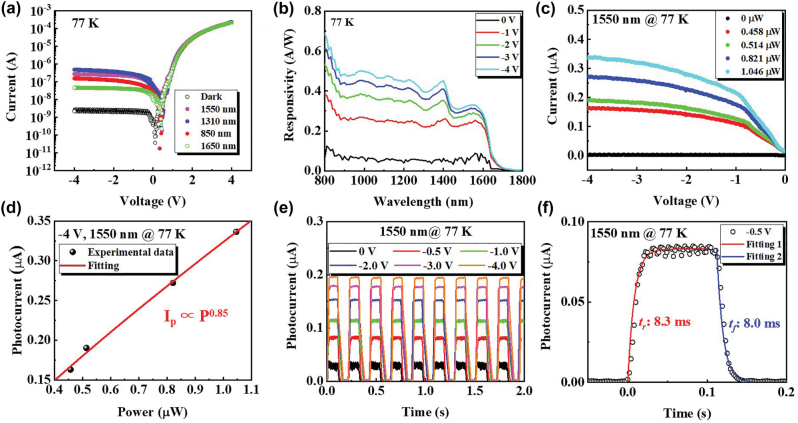
Photoresponse properties of the GeSn NW PD at 77 K. (a) Comparison of *I*–*V* curves of the GeSn NW PD under illumination of light with wavelengths of 850, 1310, 1550 and 1650 nm at 77 K. (b) Response spectra of the GeSn NW PD under biases of −4 V–0 V at 77 K. (c) Reverse *I*–*V* curves of GeSn NW PD at 77 K under 1550 nm light illumination with powers of 0 μW (dark) to 1.046 μW. (d) Dependence of photocurrent of the GeSn NW PD under 1550 nm light illumination on incident light power under −4 V at 77 K. (e) Real-time photo response of the GeSn NW PD under 1550 nm light illumination with a power of 0.514 μW under reverse biases of 0 V to −4 V. (f) Temporal response of the GeSn NW PD under single pulse of 1550 nm light with a power of 0.514 μW at 77 K under −0.5 V.

Figure 5(a) displays the *I*–*V* curves of the GeSn NW PD under illumination of light with wavelengths of 850, 1310, 1550 and 1650 nm at 77 K. The dark *I*–*V* curve is also plotted for comparison. After light illumination, the voltage at which minimum current is achieved shifts to ∼0.45 V and the reverse current dramatically increases to 46–476 nA@−4 V demonstrating evident photoresponse of the NW PD at these wavelengths.

Figure 5(b) displays the response spectra of the GeSn NW PD under 0 V to −4 V biases at 77 K. The NW PD presents a broadband response with two absorption edges: one at around 1450 nm, the other at around 1700 nm. According to Vashini’s law [46], the bandgap of Ge would increase with decreasing temperature. Under 77 K, the direct bandgap of Ge is around 0.86 eV [47]. Hence, the absorption edge near 1450 nm is determined by the direct bandgap of Ge. As Sn is incorporated into Ge, the bandgap of GeSn decreases. The absorption edge near 1700 nm can be ascribed to the cutoff absorption of GeSn direct bandgap along with quantum confinement effect. As reverse bias enlarges, the responsivity (*R*) gradually increases and saturates at large reverse bias. Under zero bias, the responsivity at 1550 nm is ∼76 mA/W. As the bias increases to −1 V, *R* at 1550 nm dramatically improves to 0.24 A/W due to broadening of the depletion region. At larger reverse bias, the NW becomes fully depleted, thus the *R* at 1550 nm gradually saturates to 0.33 A/W under −4 V. Although the volume of active region is small, the *R* of the NW PD is comparable to that of typical GeSn film PD [42]. This can be attributed to the large surface to volume ratio, strong quantum confinement effect and formation of SCG heterojunction of the NW, which enhance the interaction between light and NW and carrier confinement in the NW.

Figure 5(c) shows the reverse *I*–*V* curves of NW PD under illumination of 1550 nm light with powers of 0 μW–1.047 μW at 77 K. As light intensity increases, the photocurrent enhances apparently. Figure 5(d) summarizes the photocurrent (*I*_ph_) of the GeSn NW PD under illumination of 1550 nm light at −4 V as a function of incident light power (*P*). As *P* increases, *I*_ph_ exhibits nonlinear increments. The dependence of *I*_ph_ on *P* can be described as *I*_ph_ ∝ *P*^*α*^, where *α* is the fitting exponent. The exponent *α* is fitted to be 0.85, indicating that the incident photons can be effectively converted to photocurrent. Under incident powers of 0.458, 0.514, 0.821 and 1.046 μW, the responsivities are calculated to be 0.36, 0.37, 0.33 and 0.32 A/W, respectively. The specific detectivity (D^*^) of PD can be calculated by $D^{*}=R\sqrt{\frac{A}{2qI_{\text{dark}}}}$ , where *A* is the photosensitive surface area. Under 77 K, the D^*^ at 1550 nm of the GeSn NW PD is calculated to be ∼2.4 × 10^12^ cm Hz^1/2^ W^−1^@−4 V, which is significantly higher than the values of reported GeSn PDs at 77 K and is comparable to those of III–V PDs [42, 48]. The eminent D^*^ can be attributed to low reverse dark current and large responsivity as mentioned above. The results indicate that the fabricated self-assembled GeSn NWs is promising for low-cost nano-photoelectronic applications, such as NIR and SWIR PDs.

Figure 5(e) exhibits the real-time photo response of the GeSn NW PD under illumination of 1550 nm light with a power of 0.514 μW at 77 K under reverse biases of 0 V to −4 V. The light was periodically switched on and off using a chopper. The GeSn NW PD shows a stable and reliable response to the light pulse. Figure 5(f) further shows the temporal response of the GeSn NW PD under single pulse of 1550 nm light with a power of 0.514 μW at 77 K under −0.5 V. The rising time and falling time are extracted to be 8.3 ms and 8.0 ms, respectively, demonstrating that the NW PD has rapid switching ability.

**Figure 6:**
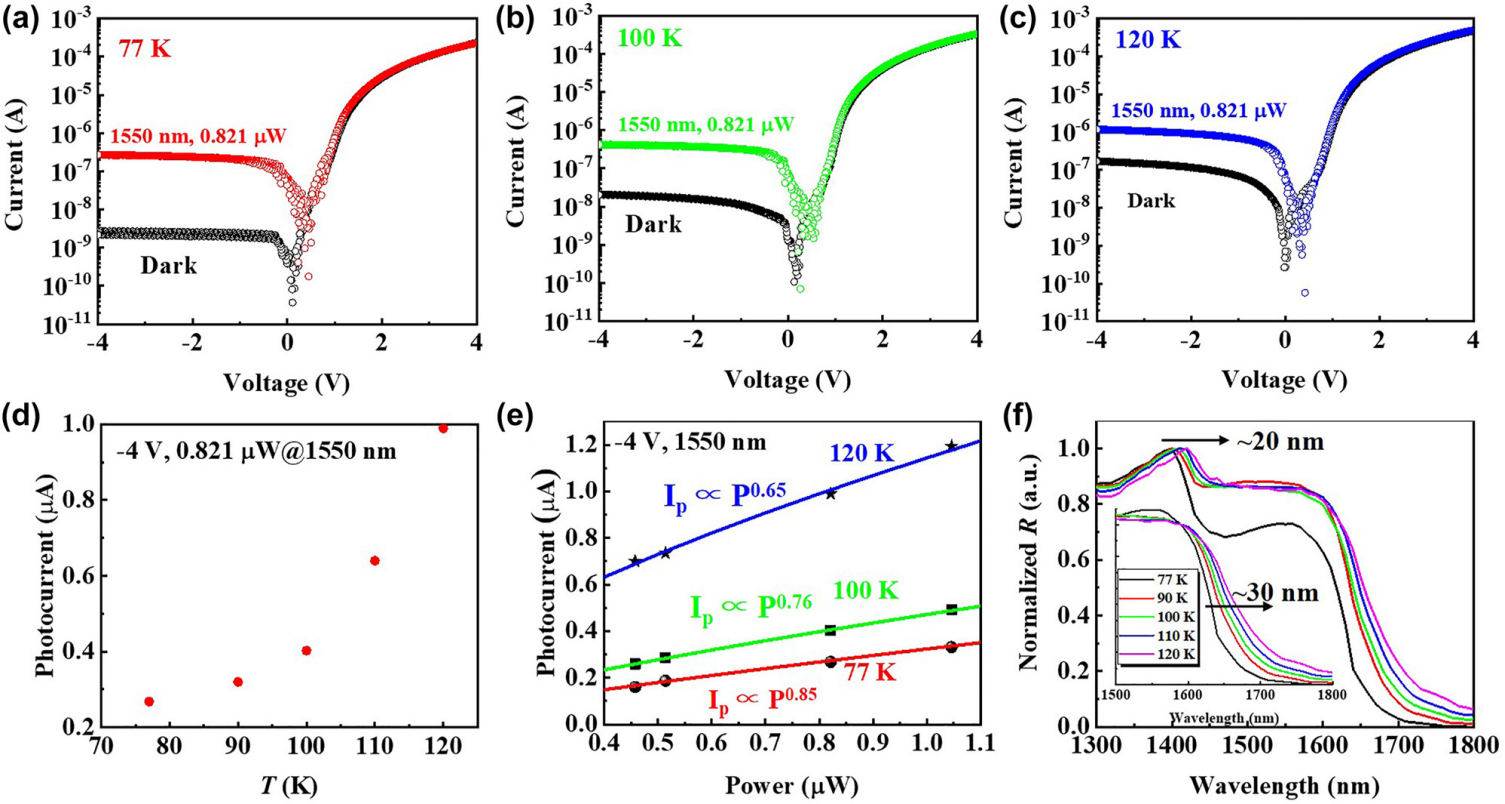
*I*–*V* curves of the GeSn NW PD under dark and illumination of 1550 nm light with a power of 0.821 μW at (a) 77 K, (b) 100 K, and (c) 120 K. (d) Photocurrent of the GeSn NW PD under illumination of 1550 nm light with a power of 0.821 μW under −4 V bias as a function of temperature. (e) Dependence of photocurrent of the GeSn NW PD under 1550 nm light illumination on incident light power under −4 V at 77 K, 100 K and 120 K. (f) Normalized response spectra of the GeSn NW PD based on Ge absorption edge and GeSn absorption edge (inset) under −4 V at 77 K–120 K.

Finally, the temperature-dependent response of the GeSn NW PD was investigated. Figure 6(a)–(c) display the *I*–*V* curves of the GeSn NW PD under illumination of 1550 nm with a power of 0.821 μW at 77 K, 100 K and 120 K, respectively. As temperature raises, the photocurrent (*I*_ph_) increases obviously suggesting enhanced responsivity. The dependence of the corresponding *I*_ph_ on temperature in the range of 77 K–120 K has been summarized in Figure 6(d). The *I*_ph_ at 120 K is enhanced to ∼3.7-fold compared to that at 77 K. The enhanced *I*_ph_ (or responsivity) can be attributed to shrinkage of Ge and GeSn bandgaps at elevated temperatures, which increase the absorption coefficient. Figure 6(e) shows the *I*_ph_ of the GeSn NW PD under illumination of 1550 nm light under −4 V as a function of incident light power (*P*) at 77 K, 100 K and 120 K. The exponent *α* at 77 K, 100 K and 120 K is fitted to be 0.85, 0.76 and 0.65, respectively. The smaller *α* at higher temperature indicate that the photogenerated electron-hole pairs can be less effectively converted to photocurrent at higher temperature. This is probably due to activation of GeSn surface shallow traps at elevated temperature, which increase the SRH recombination and can be suppressed by surface passivation techniques. Figure 6(f) compares the response spectra of the GeSn NW PD under −4 V at 77 K–120 K. The spectra have been normalized based on the responsivity at the Ge absorption edge. As temperature raise from 77 K to 120 K, a red shift of ∼20 nm of Ge response peak is observed. The inset of Figure 6(f) shows the same response spectra normalized based on the responsivity at 1600 nm. Taking the wavelength at which the responsivity drops to the half value of that at 1600 nm as reference, as the temperature raise from 77 K to 120 K, a red shift of ∼30 nm is observed for GeSn absorption edge. The red shift of Ge and GeSn absorption edge is due to shrinkage of Ge and GeSn bandgap at elevated temperature.

## 4 Conclusions

In summary, we proposed a CMOS compatible and scalable fabrication method of self-assembled GeSn vertical nanowires by RTA and ICP dry etching. After thermal treatment of MBE-grown GeSn at 550 °C, the spontaneous emission of GeSn direct band was enhanced by ∼5-fold and self-assembled Sn NDs were formed on surface. Taking the self-assembled Sn NDs as hard mask, vertical GeSn NWs were obtained by Cl-based ICP dry etching process. Using TiO_2_ as n-type transparent conductive layer, a p-GeSn NW/n-TiO_2_ prototype PD was fabricated. The electrical and optical properties of the NW PD were systematically studied. The PD exhibits an ultralow current density of ∼33 nA/cm^2^ at 77 K. At 77 K, the PD shows a responsivity of 0.245 A/W and a specific detectivity of 2.40 × 10^12^ cm Hz^1/2^ W^−1^ at 1550 nm under −1 V with a rapid switching performance. The results suggest that this method is promising for low-cost and scalable fabrication of GeSn NWs, which have a great potential for NIR or SWIR nanophotonic devices.

## Supplementary Material

Supplementary Material Details

## Supplementary Material

The article contains supplementary material (https://doi.org/10.1515/nanoph-2022-0489).
