# Supplementary material for: Scalable fabrication of self-assembled GeSn vertical nanowires for nanophotonic applications
Source: Nanophotonics. 2023 Jan 12;12(2):219–28. doi: 10.1515/nanoph-2022-0489 (PMC9889135; doi:10.1515/nanoph-2022-0489)
Supplement: Supplementary file 1 — Supplementary Material Details [file j_nanoph-2022-0489_suppl_001.docx]

**Supplementary Material for**

**“Scalable fabrication of self-assembled GeSn vertical nanowires for nanophotonic applications”**

Guangyang Lin,^1,^^[[1]](#footnote-1)^a) Yuying An,^1^ Haokun Ding,^1^ Haochen Zhao,^2^ Jianyuan Wang,^1^ Songyan Chen,^1^ Cheng Li,^1^ Ryan Hickey,^2^ James Kolodzey^2^ and Yuping Zeng^2,a)^

1. Department of Physics, Xiamen University, Xiamen, Fujian 361005, People’s Republic of China
2. Department of Electrical and Computer Engineering, University of Delaware, Newark, Delaware 19716, United States of America

**Table of contents**

1. **XRD rocking curve analysis of pseudomorphic GeSn on Ge. · · · · · · · ·· · ·2**
2. **Analysis of PL spectra taken from as-grown and annealed GeSn samples. · ·4**
3. **Modulation of GeSn nanowire size and density. · · · · · · · · · · · · · · · · 6**

**1. XRD rocking curve analysis of** **pseudomorphic GeSn on Ge.**

The rocking curve of as-grown GeSn was collected from PANalytical X’Pert PRO MRD high resolution X-ray diffractometer by performing *ω*-2*θ* scan, as shown in Fig. S1. For (004) plane, the Bragg’s diffraction condition is:

2*d*_004_sin(*θ*)=λ, (S1)

*d*_004_=*a*_⊥_/4, (S2)

where *d*_004_ is (004) interplanar spacing, *θ* is the Bragg angle between the incident beam and (004) reflecting plane, λ=0.15406 nm is the CuKα1 X-ray wavelength and *a*_⊥_ is out-of-plane lattice constant. For bulk Ge, the out-of-plane lattice constant is 0.5658 nm resulting in a Bragg angle of 33.00^o^ for ideal (004) plane. In this work, the cutting accuracy of Ge substrate surface was made within 0.5^o^. The actual offcut angle (*α*) respect to (001) plane was measured as 0.08^o^ by XRD. Based on the measured separation (*D*) between the diffraction peaks of bulk Ge and GeSn, the Bragg angle of GeSn can be calibrated by the following equation:[1]

*θ_L_*=*θ_S_*-*D*+*α*, (S3)

where *θ_L_* and *θ_S_* are Bragg angles of ideally-oriented epilayer (GeSn) and substrate (Ge), respectively. From Fig. S1, *D* was extracted as 2175 arc sec (0.6042^o^). Hence, an out-of-plane lattice constant of 0.5763 nm for GeSn can be calculated using Eqs. (S1-S3).

Thickness fringes arise when an X-ray beam is diffracted by a thin nearly perfect heteroepitaxial layer. The observation of thickness fringes in Fig. S1 indicates that GeSn is almost fully strained to bulk Ge, which means GeSn has an equal in-plane lattice constant (*a*_//_) to that of bulk Ge. The lattice constant of GeSn (*a*_GeSn_) can be then calculated by:

$a_{GeSn}=\frac{1-\nu}{1+\nu}a_{\perp}+\frac{2\nu}{1+\nu}a_{//}$， (S4)

where ν is Poisson’s coefficient of GeSn and can be obtained by linear interpolation of ν_Ge_=0.270 and ν_Sn_=0.291.[2, 3] Finally, based on Vegard’s law, the Sn content of GeSn (*x*) can be extracted by:[4]

*a*_GeSn_=(1-*x*)·*a*_Ge_+*x*·*a*_Sn_+*b*·*x*·(1-*x*), (S5)

where *a*_Ge_=0.5658 nm and *a*_Sn_=0.6489 nm are the lattice constants of bulk Ge and α-Sn,[5] respectively; *b*=0.0082 is bowing factor. Taking ν=0.271 for calculation, the lattice constant and Sn content of GeSn were deduced as 0.5718 nm and 6.62%, respectively, based on Eqs. (S4-S5).

The spacing of the fringes *δω* in the rocking curve is related to the layer thickness (*t*) by the formula:[6]

$\delta\omega=\frac{\lambda sin(\varepsilon)}{t sin(2\theta)}$, (S6)

where *ε* is the angle between the diffracted beam and the sample surface (equal to the average value of 2*θ* minus *ω* for the two fringes), 2*θ* is the average value of 2*θ* for the two fringes and *δω* is the *ω*-separation of the fringes measured in radians. To improve the result accuracy, we extracted the thickness of GeSn from 4 periods of fringe separation (the actual thickness should be four times of the calculated result). As labeled in Fig. S1, fringe peaks F1 and F2 were used for thickness calculation. The *ω*-separation of F1 and F2 was 0.1093^o^. The average value of 2*θ* and *ε* for F1 and F2 were calculated as 64.49^o^ and 34.04^o^, respectively. Based on Eq. (R6), *t*=50.11 nm can be calculated indicating a GeSn thickness of 50.11×4=200.4 nm.


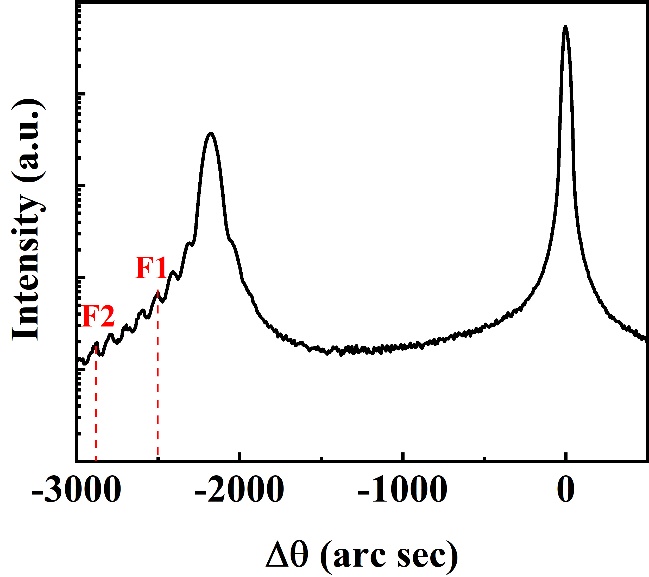


Fig. S1 Rocking curve of pseudomorphic GeSn on bulk Ge grown by MBE. Two fringe peaks F1 and F2 are labeled for GeSn thickness calculation.

**2. Analysis of PL spectra taken from as-grown and annealed GeSn samples.**

Detailed schematic of PL measurement setup is shown in Fig. S2. The as-grown sample and annealed sample were mounted on a 3-axis XYZ stage adjacent to each other at a same time for PL measurements. The PL spectra of these two samples were collected successively in the direction normal to sample surface (collected along X axis) by moving the stage along Y axis. It can be considered that the PL spectra were collected under same conditions.


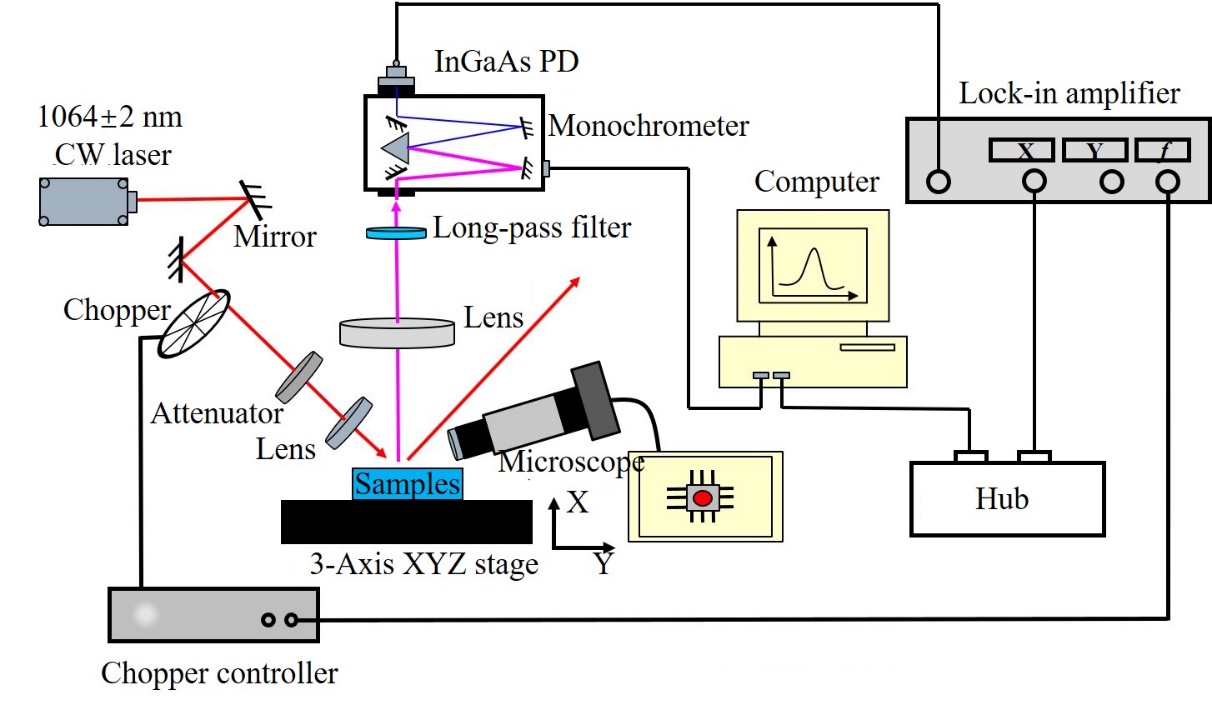


Fig. S2 Schematic of PL measurement setup. The as-grown sample and annealed sample were mounted on the 3-axis XYZ stage adjacent to each other at a same time.

We dissolved the PL spectra based on the reported results in Ref. [7]. The fitting results of PL spectra are shown in Fig. S3. The emission peak at around 1600 nm (P1) comes from direct bandgap radiation of Ge near the GeSn/Ge interface. After RTA, Sn diffuses into Ge (as confirmed by Fig. 2(i)) leading to slightly shift of P1 toward longer wavelength. The emission peaks P2 and P4 are originated from direct bandgap and indirect bandgap radiation of GeSn, respectively, agreeing well with the results reported in Ref. [8]. After annealing at 550^o^C, the emission wavelength of P2 shifts from 1934 nm to 2088 nm. Moreover, the PL intensity of P2 is improved by ~5 fold. The red shift of emission wavelength and enhanced emission intensity of P2 (GeSn direct bandgap emission) can be ascribed to strain relaxation of the GeSn film, which leads to shrinkage of GeSn bandgap and increased directness of GeSn bandgap. The result indicates that although Sn NDs are formed on GeSn surface after annealing, the optical property of GeSn is improved.


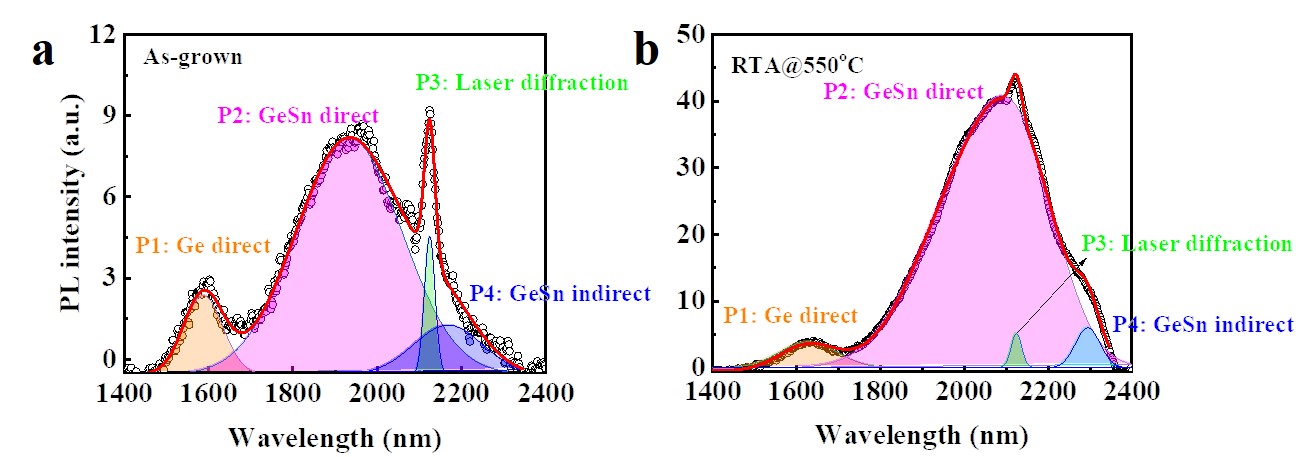


Fig. S3 Fitting results of PL spectra taken from (a) as-grown and (b) annealed GeSn samples.

**3. Modulation of GeSn nanowire size and density.**

According to our preliminary study, RTA temperature plays an important role in determining the size, height and density of the NWs. Figs. S4(a)-S4(c) exhibit the top-view SEM images of the GeSn sample after RTA at 400, 550, 600^o^C for 1 min and subsequent ICP dry etching, respectively. Figs S4(d)-S4(f) show the corresponding tilted-view SEM images, respectively. As RTA temperature rises from 400 to 550^o^C, the GeSn NW density increases from 2.2×10^8^ to 2.8×10^9^ cm^2^ since more Sn nanodots are formed on GeSn surface. As RTA temperature further rises to 600^o^C, the segregated Sn tends to form large islands resulting in a decreased NW density of 7.7×10^8^ cm^2^. For the samples obtained with RTA at 400, 550, 600^o^C, the NW diameter is 21±4 nm, 25±6 nm and 24±4 nm, the average NW height is 300 nm, 333 nm and 250 nm, respectively.


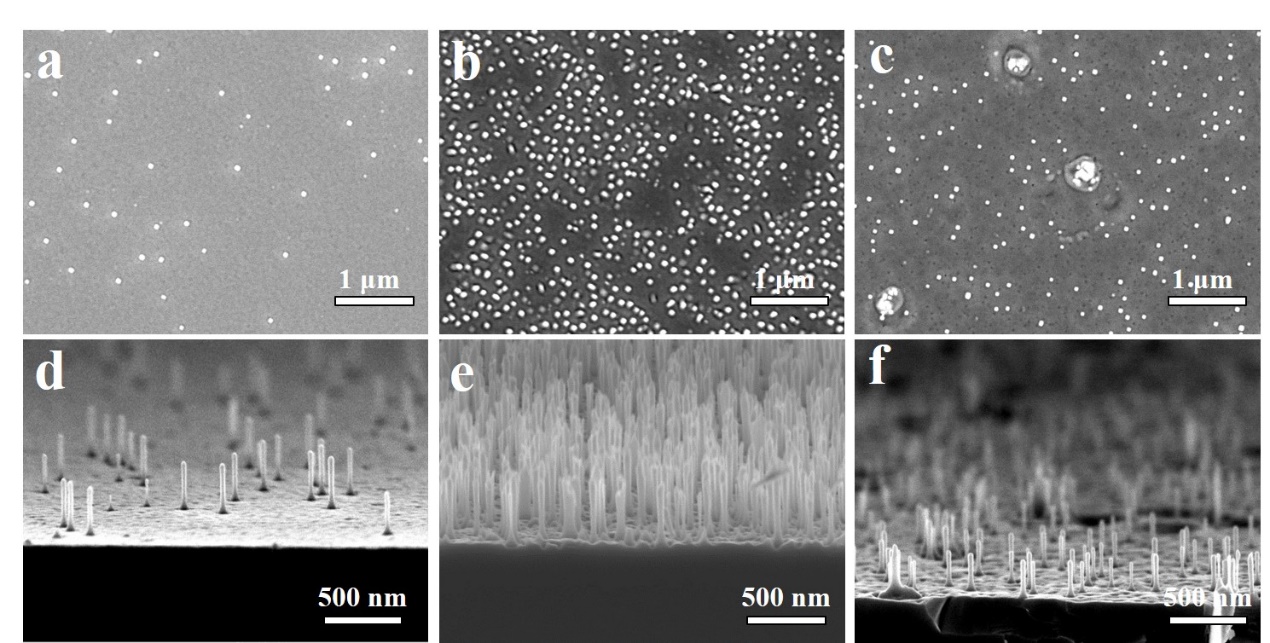


Fig. S4 (a)-(c) Top-view and (d)-(f) tilted-view SEM images of the GeSn sample after RTA at different temperatures and subsequent Cl-based ICP dry etching. (a)(d) RTA at 400^o^C; (b)(e) RTA at 550^o^C; (c)(f) RTA at 600^o^C.

Through digital etching (DE), the NW diameter can be further reduced. For example, after 1-cyle DE, which consist of O_2_ plasma treatment for 60 sec (in ICP chamber under a working pressure of 50 mTorr with RF power and ICP of 5 W and 100 W, respectively) and diluted HCl treatment (HCl:H_2_O=1:4) for 15 sec, the NW diameter obtained by RTA at 400^o^C can be further reduced to 16±2 nm, as exhibited in Fig. S5. The optoelectronic properties of the GeSn NW after DE will be studied in detail in future work.


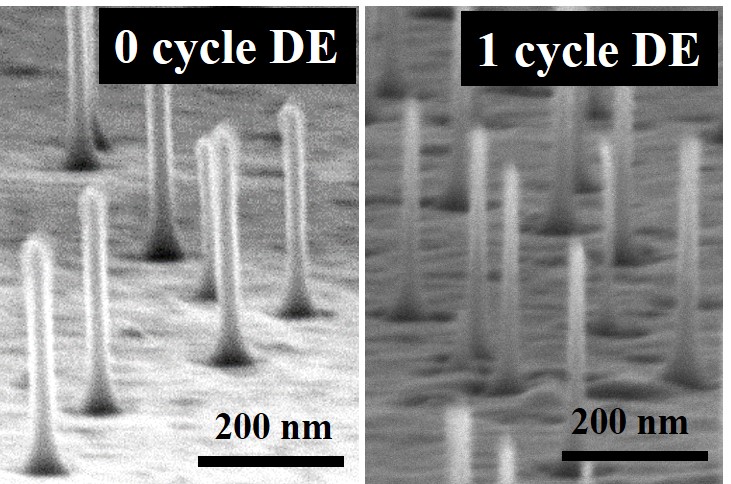


Fig. S5 Tilted-view SEM images of the GeSn NW obtained from RTA at 400^o^C and subsequent Cl-based ICP dry etching: (a) as fabricated and (b) after 1-cycle digital etching.

By reducing the dry etching time, shorter NWs can be obtained. In principle, the maximum NW height is determined by the thickness of segregated Sn nanodot, which provides a high etching selectivity over GeSn. However, further studied need to be carried out to draw a comprehensive conclusion.

**References**

1. H. Nagai, "Structure of vapor‐deposited Ga_x_In_1−x_As crystals," Journal of Applied Physics **45**, 3789-3794 (1974).

2. J. J. Wortman and R. A. Evans, "Young's Modulus, Shear Modulus, and Poisson's Ratio in Silicon and Germanium," Journal of Applied Physics **36**, 153-156 (1965).

3. S. San-Guo, "Calculation of the elastic properties of semiconductors," Journal of Physics: Condensed Matter **6**, 8733 (1994).

4. N. Bhargava, M. Coppinger, J. Prakash Gupta, L. Wielunski, and J. Kolodzey, "Lattice constant and substitutional composition of GeSn alloys grown by molecular beam epitaxy," Applied Physics Letters **103**, 041908 (2013).

5. M. Oehme, J. Werner, M. Gollhofer, M. Schmid, M. Kaschel, E. Kasper, and J. Schulze, "Room-Temperature Electroluminescence From GeSn Light-Emitting Pin Diodes on Si," IEEE Photonics Technology Letters **23**, 1751-1753 (2011).

6. "The Results Menu for Rocking Curves, Calculating Layer Thickness from Rocking Curves," in *Epitaxy software help document*.

7. S. A. Ghetmiri, W. Du, J. Margetis, A. Mosleh, L. Cousar, B. R. Conley, L. Domulevicz, A. Nazzal, G. Sun, R. A. Soref, J. Tolle, B. Li, H. A. Naseem, and S.-Q. Yu, "Direct-bandgap GeSn grown on silicon with 2230 nm photoluminescence," Applied Physics Letters **105**, 151109 (2014).

8. S. Al-Kabi, S. A. Ghetmiri, J. Margetis, W. Du, A. Mosleh, M. Alher, W. Dou, J. M. Grant, G. Sun, R. A. Soref, J. Tolle, B. Li, M. Mortazavi, H. A. Naseem, and S.-Q. Yu, "Optical Characterization of Si-Based Ge1−xSnxAlloys with Sn Compositions up to 12%," Journal of Electronic Materials **45**, 2133-2141 (2016).

1. a) Corresponding author: gylin@xmu.edu.cn and yzeng@udel.edu [↑](#footnote-ref-1)
